# Anti-NMDA receptor encephalitis and MOG-associated demyelination – a case report with long-term follow-up and a systematic review

**DOI:** 10.1186/s12883-022-02974-x

**Published:** 2022-11-16

**Authors:** Klaus Berek, Astrid Grams, Christian Uprimny, Manuela Prieschl, Melanie Ramberger, Iris Unterberger, Florian Deisenhammer, Markus Reindl, Harald Hegen

**Affiliations:** 1 Department of Neurology, Medical University of Innsbruck, Anichstrasse 35, A-6020 Innsbruck, Austria; 2 Department of Neuroradiology, Medical University of Innsbruck, Innsbruck, Austria; 3 Department of Nuclear Medicine, Medical University of Innsbruck, Innsbruck, Austria

**Keywords:** Anti-N-Methyl-D-Aspartate, NMDA, Encephalitis, Demyelination, Myelin Oligodendrocyte Glycoprotein, Case report

## Abstract

**Background:**

Overlap syndromes of anti-NMDA receptor encephalitis and MOG-mediated demyelination have been reported. In this case we provide a long-term longitudinal follow-up of clinical and imaging characteristics as well as of antibody dynamics.

**Case presentation:**

We report a 32-year-old male patient who presented with psychosis, decreased consciousness and movement disorders and was tested positive for anti-NMDA receptor antibodies. Forty-four months after symptom onset and diagnosis of autoimmune encephalitis, he suffered from relapse. At this time, the patient developed anti-MOG and anti-Caspr2 antibodies. Treatment with plasmapheresis, steroids and rituximab eventually led to substantial clinical and radiological improvement. Anti-Caspr2 antibodies persisted, anti-NMDA receptor antibodies decreased, while anti-MOG antibodies turned negative again.

**Conclusion:**

We provide long-term longitudinal follow-up of a patient with anti-NMDA receptor encephalitis who developed triple antibody positivity at the time of relapse. Antibody dynamics were associated with clinical disease course.

**Supplementary Information:**

The online version contains supplementary material available at 10.1186/s12883-022-02974-x.

## Background

Anti-N-methyl-D-aspartate-receptor (NMDAR) encephalitis and Myelin Oligodendrocyte Glycoprotein (MOG) associated disorders are both immune-mediated inflammatory disorders of the central nervous system [1, 2]. Patients with NMDAR encephalitis usually present with a clinical syndrome including psychosis, behavioural changes, amnesia and epileptic seizures, frequently followed by dyskinesia and decreased levels of consciousness [2], while patients with MOG associated disorders typically present with a demyelinating syndrome reaching from ADEM-like phenotype to isolated syndromes, such as myelitis or optic neuritis [1]. Overlap syndromes of these two disease entities have been reported, i.e. either MOG immunoglobulin G (IgG) and demyelinating aspects have been found in patients with anti-NMDAR encephalitis [3–21], or vice versa NMDAR IgG in patients with demyelinating CNS disease [22].

However, none of these reports provided long-term data of the clinical disease course, of antibody titres and radiological findings. Here, we report the case of male patient with anti-NMDAR encephalitis who developed MOG and Caspr2 IgG during the disease course.

## Case presentation

A 32-year old man with a pre-morbid modified Rankin Scale (mRS) score of 0 was referred to primary medical center due to psychosis, behavioral disorders and consecutively movement disorder, decrease in consciousness and hypoventilation. Emergency cerebral magnetic resonance imaging (MRI) revealed a normal scan. Cerebrospinal fluid (CSF) analysis showed mild pleocytosis with lymphocytes (white blood cell [WBC] count 38/μl) and CSF-restricted oligoclonal bands (OCB; Pattern II) [23]. Additionally, Computed Tomography (CT) scan and consecutive bacteriological examination of tracheal aspirate identified an atypical pneumonia due to pseudomonas aeruginosa.

The patient was referred to the Medical University of Innsbruck, where a thorough diagnostic work-up was done. Electroencephalography (EEG) showed continuous bilateral slowing without epileptic discharges. Laboratory examination by a commercially available cell based assay (Euroimmun, Lübeck, Germany) revealed positive NMDAR IgG in serum and CSF (serum titer 1:2560). Presence of IgG directed to MOG (serum titer 1:40; considered negative) and aquaporin-4 (AQP-4) were excluded by an in-house test as previously described [24, 25]. Ri, Yo, Hu, Ma2, Amphiphysin, CV2 IgG were negative (determined by a commercially available immunoblot [Euroimmun]). A whole-body 18^F^-fluoro-2-deoxy-D-glucose (18^F^-FDG) positron emission tomography (PET)/CT showed no sign of neoplastic disorder, especially no thymoma. Eventually, diagnosis of NMDAR encephalitis was made, according to the diagnostic criteria of *Graus *et al. [26].

The patient was treated with intravenous methylprednisolone (IVMP, 1000 mg on eight consecutive days) and plasmapheresis (PLEX, on four consecutive days), which led to a clinical improvement (sequelae: postencephalitic syndrome, short-term memory disturbances, apathy). Seizure occurred one month after disease onset requiring antiepileptic treatment with levetiracetam. Anti-NMDAR IgG titers declined with a nadir at month 15 after disease onset (titer 1:80). The patient further clinically improved showing a mRS score of 1 at month 15.

**Fig. 1:**
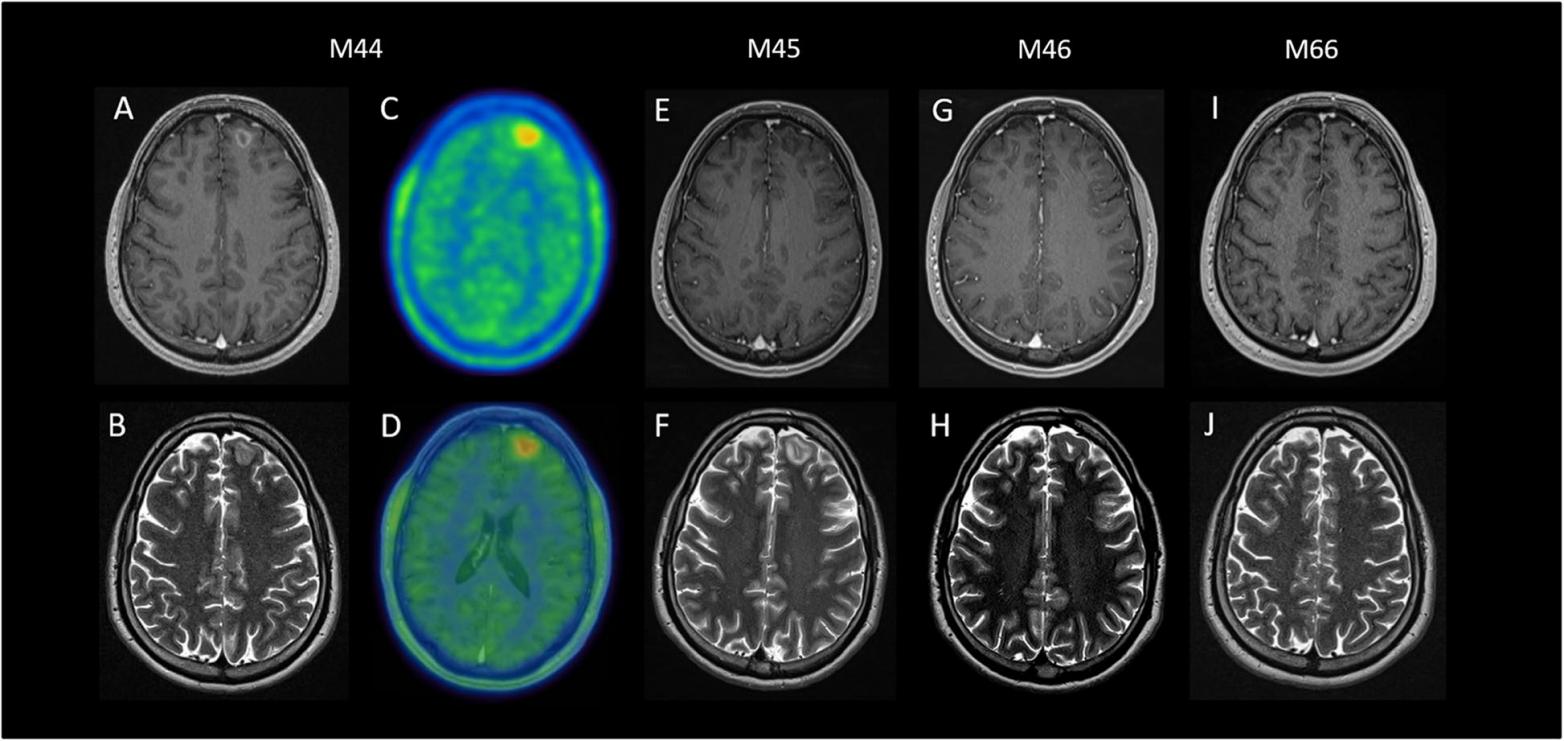
Excerpts of MRI and PET scans performed. Legend: The cerebral MRI scan during the demyelinating event at month 44 revealed a T1 hypointense, Gd enhancing (**A**), T2 hyperintense (**B**) lesion in the left Gyrus frontalis, which shows hypermetabolism in the FET-PET scan (**C**: original FET-PET image, **D**: MRI fused FET-PET image; SUV max: 2.64). MRI showed regression of lesions over time until month 66 (**E**, **G**, **I**: contrast-enhanced T1-weighted imaging. **F**, **H**,** J**: T2-weighted imaging). Abbreviations: FET-PET/CT = O-(2[18F]fluoroethyl)-L-tyrosine—positron emission tomography / computer tomography; Gd = Gadolinium; M = Month; MRI = Magnetic resonance imaging

After approximately three years of clinical remission, a slow worsening of psychiatric symptoms occurred. The patient developed new behavioral disorders, and cognitive functions declined (deterioration of short term memory, problems with word finding). CSF analysis was repeated with a WBC count of 5/μl; OCB were not detected (Pattern I) [23]. Cerebral MRI showed T2 hyperintense, T1 hypointense, Gadolinium (Gd) enhancing lesion of the white matter within the left frontal gyrus (Fig. 1A and B). This lesion showed hypermetabolism in O-(2[18F]fluoroethyl)-L-tyrosine (FET) PET (Fig. 1C and D) and reduced metabolism in 18^F^-FDG PET. Another whole-body 18^F^-FDG PET/CT revealed no sign of neoplastic disorders, especially no thymoma. EEG again showed unspecific slowing, with bilateral delta and theta activity. The laboratory work-up revealed an increase of NMDAR-IgG (serum titer 1:320). Furthermore, MOG-IgG (titer 1:320), and contactin-associated protein-like 2 (Caspr2)-IgG (as determined by a commercially available cell based assay [Euroimmun]) were found in serum (titer of 1:100), but not in CSF. Treatment with IVMP (1000 mg three days, 500 mg two days and oral tapering) initially led to a slight clinically improvement and afterwards a short stable period, however, was followed by further worsening of psychiatric symptoms after two months. Therefore, treatment with rituximab (375 mg/m^2^ body surface) was initiated at month 46, which was then followed by clinical improvement of neuropsychiatric symptoms. MRI findings and antibody titers displayed also slow regression (Fig. 1E-J).

**Fig. 2:**
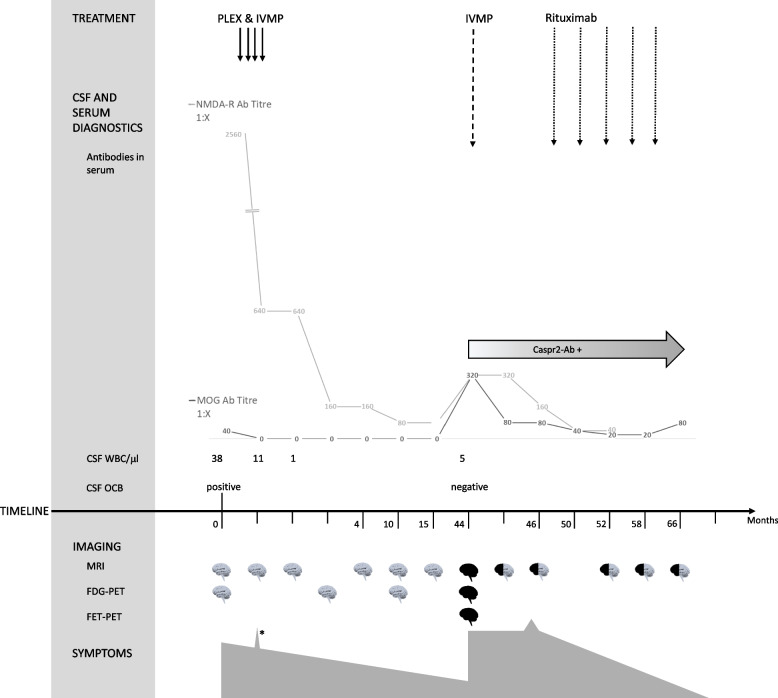
Overview of the main clinical characteristics, diagnostic results and therapeutic interventions. Legend: White “brains” indicate normal MRI findings and black “brains” indicate pathological MRI findings. White and black “brains” indicate radiological improvement. * indicates the single seizure which occurred in month 1. NMDAR IgG were detected by commercially available CBA (Euroimmun, Lübeck, Germany), titers are provided. Caspr2 IgG were detected by commercially available CBA (Euroimmun; positive at a titer of 1:10). MOG IgG were detected by an in-house CBA [24, 25], titers are provided. Abbreviations: CBA, cell-based assay PLEX = plasma exchange; IVMP = intravenous methylprednisolone; CSF = cerebrospinal fluid; WBC = white blood cells; OCB = oligoclonal bands; MRI = magnetic resonance tomography; FDG = 18^F^-fluoro-2-deoxy-D-glucose; FET = O-(2[18F]fluoroethyl)-L-tyrosine; PET = positron emission tomography; Caspr2 = contactin-associated protein-like 2; NMDAR = N-methyl-D-aspartate receptors; MOG = Myelin Oligodendrocyte Glycoprotein

Until the last follow-up at month 66 the patient had received five cycles of rituximab and showed a substantial clinical and radiological improvement (Figs. 1I and J; 2). mRS score was 1. NMDAR-IgG (at month 66) and Caspr2-IgG (at month 58) remained detectable by means of immunofluorescence until last follow-up in serum specimens, while serum MOG-IgG (at month 66) were below the cut-off titer (titer 1:80). Clinical, diagnostic and therapeutic key points are detailed in Fig. 2.

## Review of the literature

Data of clinical and radiological presentations of MOG-positive anti-NMDAR-encephalitis are still scarce and were published in form of case series and reports. We performed a systematic literature search to provide an overview on demographic, clinical, serological and radiological findings in double positive anti-NMDAR and anti-MOG patients. Therefore, we searched the medical database PUBMED on January 01, 2022 using the term “MOG” AND “NMDAR” AND “overlap” and additionally checked respective reference lists. We considered articles published since January 2000 and written in English (Supplemental Figure). A summary of our results is displayed in Table 1 and the Supplemental Table.

**Table 1:** Demographic, clinical and serological findings in MOG IgG positive patients with NMDAR encephalitis

Ref	Demographics			Clinics					Serum		CSF			
	**Patient Number**	**Age**	**Sex**	**Graus criteria fulfilled**^**a**^	**Demyelinating episodes, n**^**b**^	**Follow-up, Mo**^**c**^	**Time E – MOG + , Mo**	**DE symptoms**	**MOG-IgG status**	**NMDAR-IgG status**	**MOG-IgG status**	**NMDAR-IgG status**	**OCB status**	**WBC/µl**
[3]	1	48	F	Yes	2	60	18	Yes	positive	positive	positive	positive	positive	150
[3]	2	17	M	Yes	2	9	3	Yes	n.a	Not applicable	positive	positive	negative	3
[3]	3	27	F	Yes	5	108	-48	Yes	positive	negative	positive	positive	positive	66
[3]	4	34	M	Yes	2	22	3	Yes	positive	negative	positive	positive	negative	164
[3]	5	10	M	Yes	1	144	-96	Yes	positive	negative	positive	positive	negative	9
[3]	6	29	M	Yes	1	41	36	Yes	positive	n.a	positive	positive	negative	0
[3]	7	38	M	Yes	2	60	-22	Yes	positive	negative	positive	positive	negative	2
[3]	8	4	F	Yes	1	7	0	Yes	positive	n.a	positive	positive	positive	n.a
[3]	9	6	M	Yes	1	2,5	0	Yes	positive	negative	positive	positive	positive	43
[15]	10	9	F	Yes	1	18	0.5	Yes	positive	positive	positive	positive	negative	44
[13]	11	31	M	No	2	15	0	Yes	positive	negative	n.a	positive	negative	8
[12]	12	54	M	Yes	0	3	0	Yes	positive	n.a	n.a	positive	negative	28
[8]	13	3	F	Yes	3	252	-240	Yes	positive	negative	positive	positive	negative	0
[8]	14	23	M	Yes	3	44	-44	Yes	positive	negative	negative	positive	positive	0
[8]	15	6	M	Yes	5	54	0	Yes	positive	positive	negative	positive	positive	8
[8]	16	25	M	Yes	1	0	0	Yes	positive	negative	positive	positive	positive	96
[8]	17	9	M	Yes	2	18	18	Yes	positive	negative	negative	positive	positive	2
[11]	18	47	M	n.a	2	60	0	No	positive	positive	positive	positive	negative	16
[4]	19	36	F	Yes	1	44.5	0	No	positive	n.a	n.a	positive	positive	116
[20]	20	10	F	Yes	0	36	12	No	positive	n.a	n.a	positive	negative	576
[19]	21	6	M	Yes	0	3	0	No	positive	n.a	n.a	positive	positive	0
[18]	22	29	M	Yes	1	4	2	Yes	positive	n.a	n.a	positive	positive	39
[16]	23	12	M	Yes	0	1.5	0	No	positive	positive	n.a	positive	n.a	91
[21]	24	14	F	Yes	3	44	-4	No	positive	positive	n.a	positive	negative	256
[21]	25	10	M	Yes	0	6	0	Yes	positive	positive	n.a	positive	n.a	96
[17]	26	19	M	Yes	2	37	18	Yes	positive	negative	negative	positive	n.a	292
[6]	27	27	M	Yes	0	4	0	Yes	n.a	n.a	positive	positive	n.a	5
[10]	28	38	M	Yes	1	6	0	Yes	positive	positive	n.a	n.a	negative	88
[5]	29	37	M	Yes	0	2	0	No	positive	positive	positive	positive	negative	50
[9]	30	39	M	Yes	2	60	0	No	positive	n.a	positive	positive	n.a	112
[7]	31	30	M	Yes	0	12	0	Yes	positive	positive	n.a	positive	n.a	n.a
**Total**	**31**	**25 (3–54)**	**Female: 8 (25.8%)**	**29 (94%)**	**1 (0–5)**	**18 (0–252)**	**0 (-240–36)**	**23 (74%)**	**Positive: 29**	**Positive: 10**	**Positive: 16**	**Positive: 30**	**Positive: 11**	**43 (0–576)**

*Legend:* *CSF* Cerebrospinal fluid, *DE* Demyelination, *E* NMDAR-encephalitis, *F* Female, *IgG* Immunoglobulin G, *MO* Months, *M* Male, *MOG* Myelin oligodendrocyte glycoprotein, *MOG +* MOG IgG positivity, *N* Number, *NMDAR* N-methyl-D-aspartate receptor, *OCB* Oligoclonal bands, *Ref* Reference, *WBC* White blood cell count

^a^ Diagnostic criteria for definite NMDAR encephalitis according to Graus et al. [26]

^b^ Demyelinating episodes during or after NMDAR-encephalitis were considered

^c^ Follow-Up after NMDAR-encephalitis

C: Follow-Up after NMDAR-encephalitis

We included 31 cases from literature. The age of the predominantly male patients (male: 74.2%) ranged between 3 and 54 years, with a peak within young adulthood (median age at onset: 25). Every patient suffered in median one demyelinating event during or after NMDAR-encephalitis. Similarly, also MOG IgG were most often detected concurrently with NMDAR-encephalitis. The median time between onset of encephalitis and MOG-positivity was 0 months, ranging from -240 (indicating positive MOG IgG before onset of encephalitis) to 35 months. The detection of MOG IgG is usually linked to a clinical demyelinating event (74%). NMDAR IgG are more frequently detected within CSF, while MOG IgG are more often found in serum samples. Eleven (44%) of 25 patients tested for OCB show positive results (Pattern II or III [27]). The WBC ranged from 0 to 576, with a median of 43/µl.

Reported radiological findings of MOG IgG positive NMDAR-encephalitis patients are displayed in the Supplemental Table. Of 31 reported cases, 70 MRI scans were available. 18 were conducted during NMDAR-encephalitis, based on positive NMDAR-IgG titers and typical clinical alterations, at a time when clinical or serological (i.e. presence of MOG IgG) signs for demyelination were not present. 16 (89%) of these showed T2/ fluid-attenuated inversion recovery (FLAIR) hyperintense lesions, two (11%) Gd-enhancement and two normal results. In three cases (17%) bilateral, in 14 (78%) supratentorial and in two (11%) infratentorial lesions were detected. Lesions within the spinal cord were not described.

52 MRI scans were carried out during a demyelinating event, indicated by typical clinical symptoms and/or serological detection of anti-MOG IgG. 45 (86%) of these showed T2/FLAIR-hyperintensities, 16 (30%) Gd-enhancement and five (10%) a normal result. According the regions where lesions were found, 36 (69%) MRI scans showed supratentorial, 32 (62%) infratentorial and four (8%) spinal lesions. Bilateral lesions were described in 25 (48%) scans. So far, only one case study included an 18^F^-FDG PET/CT work-up, which showed a normal result [7]. Another study, added information about single photon emission computed tomography (SPECT), which revealed decreased technetium uptake within regions of the frontal lobe, brainstem and basal ganglia [4]. No results of FET PET/CT scans have been reported so far.

## Discussion

Overlap syndromes with the occurrence of MOG-IgG in patients with NMDAR encephalitis are a known but rare phenomenon and have been reported only in few patients [3–13, 15–21]. The present case provides a long-term follow-up over more than 5 years with detailed, longitudinally collected, clinical and radiological findings as well as antibody dynamics. Furthermore, we report triple antibody positivity with detection of Caspr2 IgG in serum at the time of relapse.

In our patient, we observed that NMDAR IgG titers declined after starting effective immunotherapy (PLEX, IVMP) concurrently with clinical amelioration, increased at the time of relapse and decreased again with treatment escalation (rituximab) and further clinical improvement. During relapse, i.e. the second episode of clinical worsening, demyelinating lesions on MRI were detected together with MOG IgG. After immune treatment of this overlap syndrome, also MOG titers and MRI findings decreased concurrently with clinical improvement.

The association of both NMDAR IgG titer with the clinical course of NMDAR encephalitis [2] and of MOG IgG titer with the clinical course of MOGAD have been reported earlier [1]. However, it still remains unclear whether IgG kinetics may mainly be caused by an intense treatment regimen (e.g. PLEX), or may in fact reflect the clinical course. It has been demonstrated that PLEX may lead to a marked reduction (of up to 97%) of autoantibodies in autoimmune encephalitis (AE), however, approximately one third of patients does not experience a clinical improvement [28].

The literature review of double positive (NMAR IgG and MOG IgG) patients revealed that in the majority of reported cases MOG IgG were detected for the first time simultaneously with the first NMDAR IgG detection (Table 1). In our patient, we detected MOG IgG only at the time of NMDAR encephalitis relapse, i.e. 44 months after the first event (Fig. 2). This might be explained by the heterogeneity of the so far reported cases of double positive patients. Reported cases subsume patients with MOG positivity before, simultaneous or after NMDAR encephalitis. However, this may also implicate a pathophysiological difference. Pathophysiology might probably be different in patients with a known MOG positive demyelinating disease and a later occurring NMDAR encephalitis [3, 8, 21] compared to patients with NMDAR encephalitis who later suffer a demyelinating relapse triggered by MOG IgG [3, 8, 15, 17, 18, 20]. Furthermore, some reported cases of simultaneous IgG positivity had a medical history of previous optic neuritis and were tested for MOG IgG for the first time at the onset of NMDAR encephalitis [11, 17, 20]. Overall, we did not find any obvious differences in our literature review of clinical, laboratory, or radiographic features between patients developing MOG IgG before or after NMDAR encephalitis.

At this point a possible limitation can be identified within the borderline positive MOG IgG titers. Indeed, titers of 1:320 have already been reported within Multiple Sclerosis and other neurological disorders. Therefore, an unspecific finding cannot be ruled out with certainty [24, 29]. From a clinical perspective, it has to be pointed out, that at relapse our patient actually presented with symptoms more consistent with encephalitis relapse rather than with a demyelinating disorder. Indeed, around 74% (23/31) of the patients in our literature review showed classical demyelinating symptoms (e.g., hemihypesthesia, diplopia, hemiparesis, vertigo) alongside with MOG IgG positivity. Most of them, 87% (20/23), revealed simultaneously T2 or FLAIR hyperintense lesions on cerebral MRI scans (two showed optic nerve enhancement). Nevertheless, the remaining 26% (8/31) of patients showed also asymptomatic lesion. Altogether, one might avoid routine MOG IgG testing in patients with NMDAR encephalitis due to the increase of false positive results, however, in patients with either a clinical or imaging findings of demyelination, MOG IgG testing might be reasonable. As patients with NMDAR encephalitis plus CNS demyelination show worse outcome, evidence of further autoimmunity might also have an impact on treatment decision.

It may be of special interest that since the time of the demyelinating relapse and occurrence of MOG IgG, our patient was tested positive for Caspr2-IgG in serum. So far, one prior case study of one patient reported a triple-positivity of anti-NMDAR, anti-MOG and anti-Caspr2-IgG, and similar to our case Caspr2-IgG remained positive until the end of follow-up [7]. A further similarity subsumes the clinical presentation, as “behavioral changes” and “episodic memory loss” were also described in the previous case report [7]. However, symptoms occurring in both, Caspr2 and NMDAR encephalitis are difficult to attribute to a distinct entity [30, 31]. MRI findings of the previously published case and of our case are contrasting, as we did not observe bilateral, cingulate and hippocampal lesions [7]. Furthermore, in our case Caspr2 IgG were detectable only in serum, whereas the other study found Caspr2 IgG also in CSF. It is known, that Caspr2 IgG may be detected in just one compartment, i.e. either in blood or CSF, and that the amount of Caspr2 IgG is usually lower in CSF than in serum [30, 32]. In general, the coincidence of IgG associated with AE (i.e. NMDAR IgG, Caspr2 IgG, etc.) has infrequently been reported before. Besides the above case report [7], a recent study screened 42,032 patients for AE IgG. In 28 patients, multiple (≥ 2) AE IgG, in two patients three and in one patient four distinct AE IgG were detected. The most common combination was Caspr2 and leucine-rich, glioma inactivated 1 (LGI1) IgG, which was revealed in 8 patients [33]. Therefore, such a double, triple or even quadruple positivity is a rare immunological phenomenon. It has been hypothesised that this phenomenon may partly be caused by a common immunologic trigger [33], which is a possibility for the above mentioned coexistence of Caspr2 and LGI1 IgG both targeting parts of the voltage-gated potassium channel complex [34]. However, in our case of a triple positivity such a common immunologic trigger is unlikely. Therefore, taking up an earlier hypothesis, it may rather be explained by a general susceptibility for autoimmune processes [35].

Nevertheless, the possibility of false positive Caspr2-IgG results has to be considered. Indeed, it has recently been reported, that the drawback of a high sensitivity of commercially CBA for the detection of Caspr2 is a higher risk for false positive results [36]. Nonetheless, we observed repeatedly positive Caspr2 IgG from relapse onset, until end of our follow-up, which excludes the possibility of false positive result, even if unspecific cross-reactivity cannot be excluded.

Within NMDAR encephalitis, typical MRI changes include white matter changes within diffusion tensor imaging (DTI), which are most often found in the cingulum and correlate with disease course [37]. However, normal MRI results even with severe clinical symptoms are a well-known phenomenon in a majority of NMDAR encephalitis patients [31, 37–39]. Patients suffering from MOG-antibody disease show normal or unspecific results in brain MRI scans in approximately two of three cases [40], although T2 hyperintensities predominantly located in infratentorial regions and brainstem can be found [41].

Regarding radiological findings in double positive patients we provide a review of the literature within Supplemental Table, from where it may be deduced that MRI scans at the time of demyelination show more frequently Gd-enhancing, bilateral, infratentorial and spinal cord lesions. In our patient, all MRI scans during the first, non-demyelinating episode revealed normal results, while MRI scans during the demyelinating relapse yielded T2 hyper-, T1 hypointense, Gd-enhancing lesions in the gray matter of the left Gyrus frontalis. Similar findings (i.e. normal MRI during NMDAR encephalitis and T2 hyperintense lesions during a second demyelinating event) have been reported in 11% of the cases. The radiological follow-up of our patient revealed remission of Gd-enhancement one month after treatment initiation and regression of T2-hyperintense lesions subsequently. In the last MRI scan at month 66, only subtle sequelae in form of a post-inflammatory gliosis in the left frontal lobe was found. Taking into account serological findings of our patient an association of MOG-IgG with MRI findings may be hypothesized, as negative MOG-IgG titers were found simultaneously with normal MRI findings and decreasing MOG-IgG titers were found simultaneously with receding MRI findings.

A further novelty of our case is detailed information about 18^F^-FDG PET and FET PET within double positive anti-NMDAR and anti-MOG patients. Published data are subsumed in the Supplemental Table. Previous studies have already reported that in NMDAR encephalitis 18^F^-FDG PET can reveal both hypo- and hypermetabolism [42]. With our case, we contribute longitudinal data of four 18^F^-FDG PET scans and one FET PET scan. During the first, non-demyelinating episode no pathological finding was detectable in the brain. At the time of the demyelinating relapse, FET PET detected hypermetabolism in the areas of T2 hyperintensity of the corresponding MRI scans, whereas 18^F^-FDG PET showed a diminished metabolism within these areas.

## Conclusion

We provide detailed longitudinal data of a patient with NMDAR encephalitis. At a second event, he presented with demyelinating brain lesions and MOG IgG as well as Caspr2-IgG. NMDAR and MOG IgG titers diminished alongside with clinical and radiological amelioration after IVMP, PLEX and rituximab treatment. Furthermore, we provide a systematic review regarding MOG IgG positive NMDAR encephalitis.

## Supplementary Information

**Additional file 1: Supplemental Figure. **Flowchart of included articles.

**Additional file 2: Supplemental Table. **Radiological findings in MOG IgG positive patients with NMDAR encephalitis.

## Data Availability

The datasets used and/or analysed during the current study are available from the corresponding author on reasonable request.

## Abbreviations

18^F^-FDG: 18^F^-fluoro-2-deoxy-D-glucose
AE: Autoimmune encephalitis
AQP-4: Aquaporin-4
Caspr2: Contactin-associated protein-like 2
CBA: Cell-based assay
CSF: Cerebrospinal fluid
CT: Computed Tomography
DTI: Diffusion tensor imaging
EEG: Electroencephalography
FET: O-(2[18F]fluoroethyl)-L-tyrosine
FLAIR: Fluid-attenuated inversion recovery
IVMP: Intravenous methylprednisolone
MOG: Myelin Oligodendrocyte Glycoprotein
MRI: Magnetic resonance imaging
mRS: Modified Rankin Scale
NMDAR: N-methyl-D-aspartate-receptor
OCB: Oligoclonal bands
OPS: Organic psycho syndrome
PET: Positron emission tomography
PLEX: Plasmapheresis
WBC: White blood cell
