# Supplementary figures and images for: Anti-NMDA receptor encephalitis and MOG-associated demyelination – a case report with long-term follow-up and a systematic review
Source: BMC Neurol. 2022 Nov 16;22:434. doi: 10.1186/s12883-022-02974-x (PMC9667590; doi:10.1186/s12883-022-02974-x)

**Supplemental Figure:** Flowchart of included articles

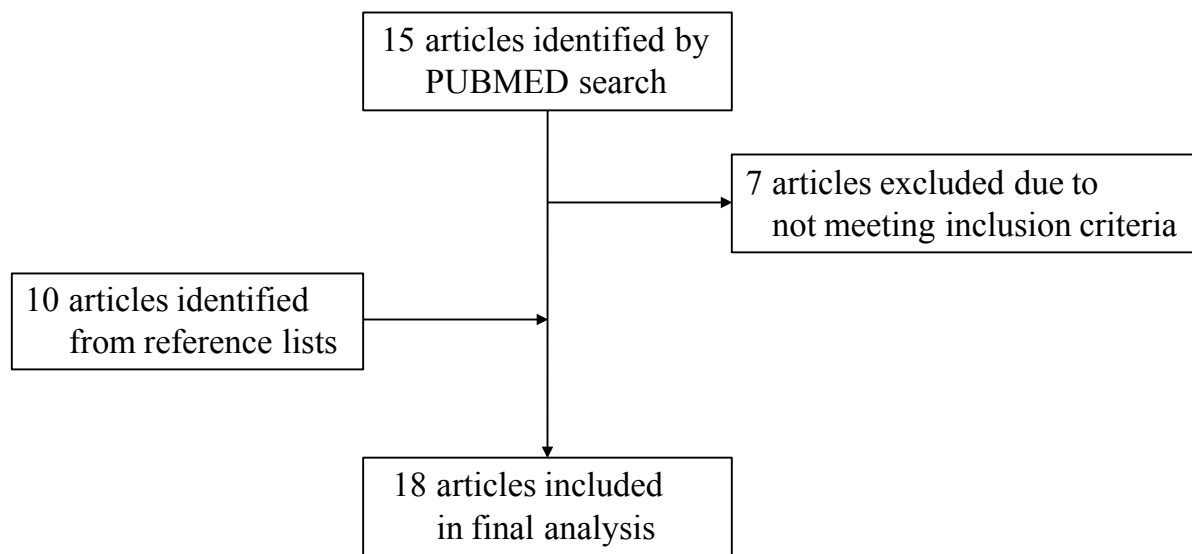

Supplement: Supplementary file 1 — Additional file 1: Supplemental Figure. Flowchart of included articles. [file 12883_2022_2974_MOESM1_ESM.pdf]
