# Supplementary material for: Anti-NMDA receptor encephalitis and MOG-associated demyelination – a case report with long-term follow-up and a systematic review
Source: BMC Neurol. 2022 Nov 16;22:434. doi: 10.1186/s12883-022-02974-x (PMC9667590; doi:10.1186/s12883-022-02974-x)
Supplement: Supplementary file 2 — Additional file 2: Supplemental Table. Radiological findings in MOG IgG positive patients with NMDAR encephalitis. [file 12883_2022_2974_MOESM2_ESM.pdf]

Supplemental Table: Radiological findings in MOG IgG positive patients with NMDAR encephalitis

Ref	Patient Number	MRI findings								PET/SPECT normal, n / abnormal, n
		Number of MRI scans	Normal, n	Presence of T2 hyperintense lesions, n	Presence of CE lesions, n	Presence of bilateral lesions, n	Presence of supratentorial lesions	Presence of infratentorial lesions, n	Presence of spinal lesions, n	
(1)	1	3	0	3	1	1	2	2	0	n.a.
(1)	2	3	0	3	0	1	2	2	1	n.a.
(1)	3	4	0	4	0	1	2	3	0	n.a.
(1)	4	3	0	3	0	2	2	3	1	n.a.
(1)	5	2	0	2	1	1	2	0	0	n.a.
(1)	6	2	0	1	2	1	2	0	0	n.a.
(1)	7	3	0	3	0	0	3	2	0	n.a.
(1)	8	1	0	1	0	1	1	1	0	n.a.
(1)	9	1	0	1	1	1	1	1	1	n.a.
(2)	10	2	0	1	1	1	2	1	0	n.a.
(3)	11	2	0	2	0	0	1	1	0	n.a.
(4)	12	1	0	1	0	1	1	1	1	n.a.
(5)	13	2	0	2	1	2	2	1	0	n.a.
(5)	14	3	0	3	1	1	2	3	0	n.a.
(5)	15	5	0	5	0	4	5	3	0	n.a.
(5)	16	1	0	1	0	0	1	0	0	n.a.
(5)	17	2	1	1	0	0	1	0	0	n.a.
(6)	18	2	1	1	0	0	1	0	0	n.a.
(7)	19	2	0	2	1	2	2	1	0	0 / 1[a]

(8)	20	3	0	3	2	1	3	0	0	n.a.
(9)	21	1	1	0	0	0	0	0	0	n.a.
(10)	22	3	0	3	0	0	2	1	0	n.a.
(11)	23	2	0	2	0	2	2	0	0	n.a.
(12)	24	3	0	3	1	1	1	2	0	n.a.
(12)	25	1	0	1	1	0	1	0	0	n.a.
(13)	26	2	2	2	0	0	1	2	1	n.a.
(14)	27	1	0	1	0	1	1	1	0	n.a.
(15)	28	3	0	3	3	1	1	3	0	n.a.
(16)	29	2	1	1	1	1	1	0	0	n.a.
(17)	30	1	1	1	0	1	1	0	0	n.a.
(18)	31	1	0	1	1	1	1	1	0	1 / 0
**Total**	**31**	**70**	**7 (10%)**	**61 (87%)**	**18 (26%)**	**28 (40%)**	**50 (71%)**	**34 (49%)**	**4 (6%)**	**1 / 1[a]**

Legend:

[a] SPECT showed reduced uptake (marker: technetium-99m ethyl cysteinate dimer) in the medial aspect of the frontal lobe, brainstem and basal ganglia.

CE = Contrast enhancing; DE = Demyelination; E = NMDAR-encephalitis; N = Number; OCB = Oligoclonal bands; PET = Positron emission tomography; Ref = Reference; SPECT = Single photon emission computed tomography.

References

1. Titulaer MJ, Höftberger R, Iizuka T, Leypoldt F, McCracken L, Cellucci T, et al. Overlapping demyelinating syndromes and anti–N-methyl-D-aspartate receptor encephalitis. Ann Neurol. 2014;75(3):411-28.
2. Yokoyama K, Hori M, Yoshida A. Anti-myelin oligodendrocyte glycoprotein antibody neuritis optica following anti-NMDA receptor encephalitis. Pediatr Int. 2016;58(9):953-4.
3. Zhou L, ZhangBao J, Li H, Li X, Huang Y, Wang M, et al. Cerebral cortical encephalitis followed by recurrent CNS demyelination in a patient with concomitant anti-MOG and anti-NMDA receptor antibodies. Mult Scler Relat Disord. 2017;18:90-2.
4. Zhou J, Tan W, Tan SE, Hu J, Chen Z, Wang K. An unusual case of anti-MOG CNS demyelination with concomitant mild anti-NMDAR encephalitis. J Neuroimmunol. 2018;320:107-10.
5. Fan S, Xu Y, Ren H, Guan H, Feng F, Gao X, et al. Comparison of myelin oligodendrocyte glycoprotein (MOG)-antibody disease and AQP4-IgG-positive neuromyelitis optica spectrum disorder (NMOSD) when they co-exist with anti-NMDA (N-methyl-D-aspartate) receptor encephalitis. Mult Scler Relat Disord. 2018;20:144-52.
6. Rojc B, Podnar B, Graus F. A case of recurrent MOG antibody positive bilateral optic neuritis and anti-NMDAR encephalitis: Different biological evolution of the two associated antibodies. J Neuroimmunol. 2019;328:86-8.
7. Aoe S, Kokudo Y, Takata T, Kobara H, Yamamoto M, Touge T, et al. Repeated anti-N-methyl-D-aspartate receptor encephalitis coexisting with anti-myelin oligodendrocyte glycoprotein antibody-associated diseases: A case report. Mult Scler Relat Disord. 2019;35:182-4.
8. Taraschenko O, Zabad R. Overlapping demyelinating syndrome and anti-N-methyl-d-aspartate receptor encephalitis with seizures. Epilepsy Behav Rep. 2019;12:100338.
9. Sarigecili E, Cobanogullari MD, Komur M, Okuyaz C. A rare concurrence: Antibodies against Myelin Oligodendrocyte Glycoprotein and N-methyl-d-aspartate receptor in a child. Mult Scler Relat Disord. 2019;28:101-3.
10. Perez CA, Agyei P, Gogia B, Harrison R, Samudralwar R. Overlapping autoimmune syndrome: A case of concomitant anti-NMDAR encephalitis and myelin oligodendrocyte glycoprotein (MOG) antibody disease. J Neuroimmunol. 2020;339:577124.
11. Ma J, Jiang L. Viral encephalitis followed by anti-NMDAR encephalitis with concomitant MOG antibody-positive central nervous system demyelination in a child. Neurol Sci. 2020;41(8):2303-5.
12. Wegener-Panzer A, Cleaveland R, Wendel EM, Baumann M, Bertolini A, Hausler M, et al. Clinical and imaging features of children with autoimmune encephalitis and MOG antibodies. Neurol Neuroimmunol Neuroinflamm. 2020;7(4).
13. Nan D, Zhang Y, Han J, Jin T. Clinical features and management of coexisting anti-N-methyl-D-aspartate receptor encephalitis and myelin oligodendrocyte glycoprotein antibody-associated encephalomyelitis: a case report and review of the literature. Neurol Sci. 2021;42(3):847-55.
14. Caparo-Zamalloa C, Alvarez-Toledo K, Yamunaque-Chunga C, Castro-Suarez S, Guevara-Silva E, Osorio-Marcatinco V, et al. Autoimmune neurology: Co-occurrence of anti-NMDAR encephalitis and anti-MOG associated disease, report of a case. J Neuroimmunol. 2021;358:577663.
15. Ren BY, Guo Y, Han J, Wang Q, Li ZW. Case Report: Anti-NMDAR Encephalitis With Anti-MOG CNS Demyelination After Recurrent CNS Demyelination. Front Neurol. 2021;12:639265.
16. Cao L, Ren L, Huang X. Clinical analysis of a patient simultaneously positive for antibodies of myelin oligodendrocyte glycoprotein and anti-N-methyl-D-aspartate receptor: A case report. Medicine (Baltimore). 2021;100(1):e24234.
17. Fujimori J, Takahashi T, Kaneko K, Atobe Y, Nakashima I. Anti-NMDAR encephalitis may develop concurrently with anti-MOG antibody-associated bilateral medial frontal cerebral cortical encephalitis and relapse with elevated CSF IL-6 and CXCL13. Mult Scler Relat Disord. 2021;47:102611.
18. Cherian A, Divya KP, Shetty SC, Kannoth S, Thomas B. Coexistent MOG, NMDAR, CASPR2 antibody positivity: Triumph over the triumvirate. Mult Scler Relat Disord. 2020;46:102468.
